# Comparative Genomics of Clinical Isolates of the Emerging Tick-Borne Pathogen *Neoehrlichia mikurensis*

**DOI:** 10.3390/microorganisms9071488

**Published:** 2021-07-13

**Authors:** Anna Grankvist, Daniel Jaén-Luchoro, Linda Wass, Per Sikora, Christine Wennerås

**Affiliations:** 1 Department of Clinical Microbiology, Sahlgrenska University Hospital, 413 46 Gothenburg, Sweden; linda.wass@vgregion.se (L.W.); christine.wenneras@gu.se (C.W.); 2 Department of Infectious Diseases, Sahlgrenska Academy, University of Gothenburg, 413 46 Gothenburg, Sweden; daniel.jaen.luchoro@gu.se; 3 Department of Pathology and Genetics, Institute of Biomedicine, Sahlgrenska Academy, University of Gothenburg, 413 46 Gothenburg, Sweden; per.sikora@gu.se; 4 Clinical Genomics Gothenburg, SciLife Labs, 413 46 Gothenburg, Sweden

**Keywords:** whole-genome sequencing, de novo sequencing, *Anaplasmataceae*, *Candidatus* Neoehrlichia mikurensis, human, neoehrlichiosis

## Abstract

Tick-borne ‘*Neoehrlichia (N.) mikurensis*’ is the cause of neoehrlichiosis, an infectious vasculitis of humans. This strict intracellular pathogen is a member of the family *Anaplasmataceae* and has been unculturable until recently. The only available genetic data on this new pathogen are six partially sequenced housekeeping genes. The aim of this study was to advance the knowledge regarding ‘*N. mikurensis*’ genomic relatedness with other *Anaplasmataceae* members, intra-species genotypic variability and potential virulence factors explaining its tropism for vascular endothelium. Here, we present the de novo whole-genome sequences of three ‘*N. mikurensis*’ strains derived from Swedish patients diagnosed with neoehrlichiosis. The genomes were obtained by extraction of DNA from patient plasma, library preparation using 10× Chromium technology, and sequencing by Illumina Hiseq-4500. ‘*N. mikurensis*’ was found to have the next smallest genome of the *Anaplasmataceae* family (1.1 Mbp with 27% GC contents) consisting of 845 protein-coding genes, every third of which with unknown function. Comparative genomic analyses revealed that ‘*N. mikurensis*’ was more closely related to *Ehrlichia chaffeensis* than to *Ehrlichia ruminantium*, the opposite of what 16SrRNA sequence-based phylogenetic analyses determined. The genetic variability of the three whole-genome-sequenced ‘*N. mikurensis*’ strains was extremely low, between 0.14 and 0.22‰, a variation that was associated with geographic origin. No protein-coding genes exclusively shared by *N. mikurensis* and *E. ruminantium* were identified to explain their common tropism for vascular endothelium.

## 1. Introduction

‘*Candidatus (Ca.)* Neoehrlichia (N.) mikurensis’ was first isolated in ticks and wild rodents on the Japanese island of Mikura in 2004 [1]. In Europe, it is one of the most common human-pathogenic microbes carried by *Ixodes ricinus* ticks, after *Borrelia burgdorferi* sensu lato and *Rickettsia spp.* [2,3,4]. The bacterium gained attention in 2010, when several case reports revealed its capacity to cause human disease [5,6,7,8], which was later named as neoehrlichiosis [9]. Severe cases of neoehrlichiosis typically feature high fever with thromboembolic or vascular complications, [9] which is a consequence of ‘*Ca*. N. mikurensis’ tropism for vascular endothelium [10]. Initially, ‘*Ca*. N. mikurensis’ was considered an opportunistic bacterium that chiefly afflicted immune-suppressed patients with particular hematologic or autoimmune diseases [9]. However, it is increasingly appreciated that persons with normal immune defenses can also become infected by this new pathogen and presented with disease manifestations ranging from asymptomatic infection, skin rash, systemic infection with fever and even suspected death from vascular complications [6,11,12,13]. The bacterium has been considered unculturable until recently, when we were able to cultivate it in tick cell lines and human primary endothelial cell lines [10].

‘*Candidatus* Neoehrlichia’ is the latest genus to be described out of the 7 genera currently comprising the family *Anaplasmataceae*. ‘*Candidatus* Neoehrlichia lotoris’ was the first species to be identified within this genus and is carried by North American raccoons and their associated tick species [14]. Previous studies of the genetic diversity of ‘*Ca*. N. mikurensis’ based on sequencing of housekeeping genes, particularly the 16S rRNA and *groEL* genes, indicated that ‘*Ca*. N. mikurensis’ is closely related to *Ehrlichia ruminantium*, less so to *Anaplasma phagocytophilum* and least of all to *Wolbachia endosymbiont* [6,8,15,16,17].

Three genotypes of ‘*Ca*. N. mikurensis’ were identified amongst 12 European human isolates analyzed by Multilocus sequence assay [MLSA]: one from the western part of Sweden, one from central Europe and a pan-European type [17]. Overall, there was low genetic diversity in the six analyzed MLSA loci, indicating that the strains infecting humans in Europe were quite similar [17]. In contrast, comparative alignment of the 16S rRNA and *groEL* gene sequences of European and Asian ‘*Ca*. N. mikurensis’ strains revealed that they differed considerably from each other [6,15,17].

The main objective of this study was to sequence the entire genome of ‘*Ca*. N. mikurensis’ to increase the knowledge regarding this emerging pathogen. Specifically, we wanted to shed light on the relatedness of this pathogen with other bacterial species within the *Anaplasmataceae* family, and possibly unravel shared genes with bacterial species having documented tropism for vascular endothelium, e.g., *Ehrlichia ruminantium* [18]. Complete sequences of five previously sequenced MLSA genes (16S rRNA, *ftsZ*, *gatB*, *groEL* and *lipA*) as well as fragments evaluate the degree of intra-species variability between different clinical isolates of ‘*Ca*. N. mikurensis’. Our strategy was to attempt whole-genome sequencing using two types of material: experimentally infected tick cell lines and plasma samples from neoehrlichiosis patients not yet treated with antibiotics.

## 2. Materials and Methods

### 2.1. Clinical Isolates of Ca. N. mikurensis

EDTA-anticoagulated blood samples from three Swedish immunocompromised neoehrlichiosis patients (patient and strain ID SE20, SE24 and SE26) were used. Clinical data pertaining to these patients have been published previously [10]. Neoehrlichiosis was diagnosed by PCR using plasma from EDTA-blood as follows: a real-time PCR against the *groEL* gene of ‘*Ca*. N. mikurensis’ was run first, and subsequently confirmed by pan-bacterial PCR reactions with Sanger sequencing of the 16S rRNA gene [11].

### 2.2. Tick Cell Line Cultivated Isolate of Ca. N. mikurensis

The embryo-derived tick cell line IRE/CTVM20 was inoculated with whole blood from a patient (SE18), diagnosed with neoehrlichiosis, and incubated for 21 weeks according to a published protocol [10]. Successful infection of the tick cells was confirmed as previously described [10,11]. Briefly, visualization of the bacteria inside the tick cells was achieved by image-flow cytometry, as well as by quantification of bacterial DNA in tick cell cultures by the *groEL*-based real-time PCR.

### 2.3. Bacterial DNA Extraction

The MagAttract HMW DNA Kit (Qiagen, Hilden, Germany) was used for purification of high-molecular-weight DNA from patient plasma and tick cells, using magnetic beads according to the manufacturer’s protocol. DNA yields and purity were measured by automated electrophoresis using a Tape Station and gDNA kit (Agilent Technologies, Santa Clara, CA, USA).

### 2.4. 10X Chromium Library and Sequencing

10X Chromium Technology (10X Genomics, Pleasanton, CA, USA) was applied for Gel Bead-In Emulsion (GEM) library preparations (Chromium Genome 10X Library kit, 10X Genomics) using 0.7–1.3 ng/µL of HMW-extracted DNA according to the manufacturer’s recommendations. Final library DNA concentrations and DNA fragment sizes were determined by Tape Station electrophoresis as described above. The libraries were sequenced at Clinical Genomics Stockholm, SciLife Labs, using an Illumina HighSeq-4500 platform in two runs. The first run was based on a fresh blood sample (patient ID SE24) and was selected to be a proof-of-concept sequence run. The subsequent runs were based on two additional frozen plasma preparations from the same patient (SE24-1 and SE24-2) and two additional patient samples, SE20 and SE26. The SE24 libraries were assembled using a combination of Supernova (Supernova v.2.0.1 assembler) [19] using standard parameters, BLAST (GenBank BLAST program http://blast.ncbi.nlm.nih.gov/Blast.cgi, accessed on 11 April 2019) and CLC Genome Finishing module (CLC bio, Aarhus, Denmark). Genomes were finally annotated with Prokka v1.11 software [20]. The first draft assembly was created by initial mapping of the entire SE24 data set against a human whole-genome reference (HG19) and collecting any reads that did not map to the reference [21]. After assembly, the SE20 and SE26 libraries were mapped against the SE24 assembly using CLC Genomics Workbench software with default settings, followed by a fixed ploidy variant calling (90% probability, 80% minimum frequency, minimum coverage 10, minimum count 8, filter homopolymers >3). Lastly, the genome sequences were annotated with the Prokaryotic Genome Annotation Pipeline [22,23] and submitted to GenBank.

### 2.5. Genomic Analyses and Comparisons

Bioinformatic analyses to categorize the functions of the proteins encoded by the sequenced *Ca*. N. mikurensis genomes were done using eggNOG Mapper v2 [24,25].

Pan-genome analyses were also done using the type-strain genomes of *Ehrlichia chaffeensis* Arkansas^T^, *Ehrlichia ruminantium* Welgevonden^T^, the strain *Anaplasma phagocytophilum* HZ (the first published genome of *A. phagocytophilum* [26]) and the genomes of reference strains ‘*Ca*. N. lotoris’ RAC-413 and ‘*Ca*. N. mikurensis’ SE24. For normalization purposes, all genomes were annotated using Prokka v1.11. The proteins sequences of the genomes were compared (all vs. all) using the Basic Local Alignment Search Tool for Proteins (BLASTP) [27]. Based on these results, groups of homologous proteins were formed, using the Get Homologues software [28] and based on two different algorithms: Cluster of Orthologous Genes Triangle (COGT) [29] and Orthologous Markov Cluster (OMCL) [30]. The threshold for homology was set to 70% similarity for at least 70% of the respective sequence [31]. Only clusters of homologous proteins detected by both algorithms were considered for further characterizations. A Venn Diagram was constructed based on the presence and absence of the different clusters among the species studied to compare the numbers of shared proteins.

The presence or absence of prophages was determined using the online tool Prophage Hunter [32]. The results are classified by this tool as “Active” (score 0.8–1) or “Ambiguous” (score 0.5–0.8). Functional categorizations of the sets of proteins extracted from the genomes were performed with eggNOG-Mapper v2 [24,25].

### 2.6. Phylogenetic Analyses

Complete sequences of the16S rRNA genes were extracted from the whole-genome sequences of the strains listed above. Sequences were aligned and similarity matrices were generated. Evolutionary distances were calculated using the Kimura two-parameter model [33]. Clustering analysis was performed and a phylogenetic tree was built based on neighbor-joining [34] using the MEGA v7 software [35]. Bootstrap was set for 1000 replications. Additionally, a core-genome phylogenetic analysis was done, using the same genome annotations employed in the previous pan-genome determination. BLASTP analyses comparing all protein sequence files were made. Based on these results, homologous searches were done using the algorithms COGT, OMCL, Bi-Directional Best Hits (BDBH) [36] and Get_Homologues software. The homology threshold was set as explained previously (70% similarity in at least 70% of the sequences). A core genome composed of proteins encoded by single-copy genes was determined based on the consensus reached by the three algorithms. Each protein group was aligned using Clustal Omega [37]. Alignments were analyzed by GBLOCKS [38] to generate a concatenation of the regions with homologous positions. This final alignment was used to build a core genome tree using the Maximum Likelihood algorithm [39] and the Approximate Likelihood Ratio Test (aLRT) [40] with PhyML software [41].

## 3. Results and Discussion

**Figure 1:**
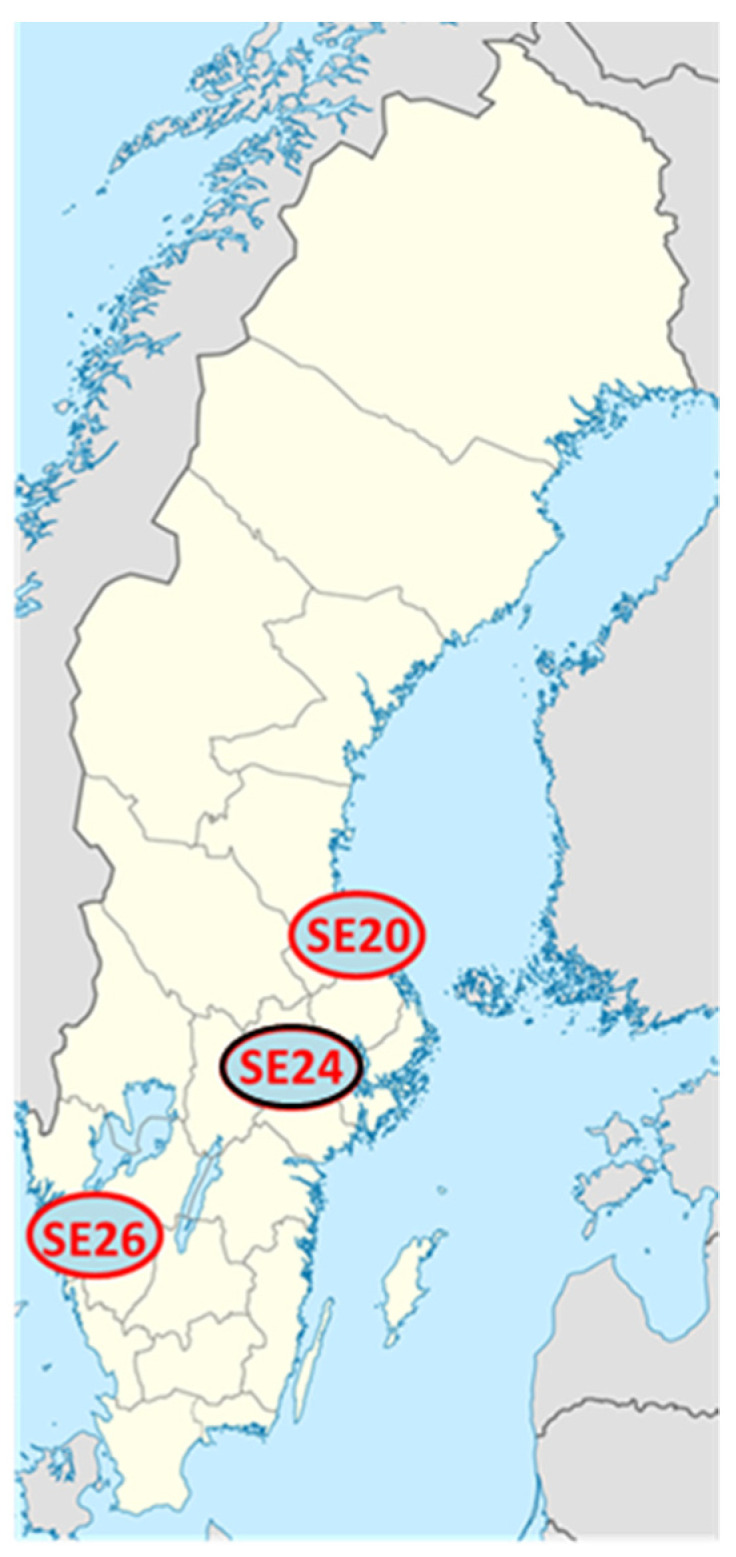
Geographic origin of the sequenced human *Ca*. N. mikurensis isolates. The sites of residence of the three patients diagnosed with neoehrlichiosis whose blood samples were sequenced are shown. Strain ID SE20 = Gävle, SE24 = Eskilstuna (reference genome, marked in black), SE26 = Kungälv.

Here, we present the complete genome sequence and genomic features of the reference strain *Ca*. N. mikurensis SE24 (GenBank accession no. CP066557), a clinical isolate from a patient diagnosed with neoehrlichiosis. We also present the genome sequences of two additional Swedish clinical isolates: *Ca*. N. mikurensis, SE20 (GenBank accession No. CP054597) and SE26 (GenBank accession No. CP060793). We selected clinical isolates derived from immune-suppressed neoehrlichiosis patients because they usually have several 10-log higher concentrations of bacterial DNA in the blood compared with immune-competent subjects [9]. All three *Ca*. N. mikurensis strains were derived from patients from different geographic locations in Sweden (Figure 1).

The sequencing of these clinical isolates from patient plasma was successful despite the low fractions of bacterial DNA, ranging from 0.1 to 5.1% of the total extracted DNA (Table 1). Meanwhile, enrichment for bacterial DNA by propagating the infection from patient plasma onto tick cells only generated sequence data belonging to *Ixodes ricinus*. A possible explanation for this failure may be that the bacteria were harvested too late during the infection and, although the tick cells were massively infected, the bacteria may have started to die, thus yielding poor-quality DNA. Moreover, the successful sequencing outcome when using human plasma directly may have depended on the fact that contamination of bacterial DNA with human DNA is advantageous when using 10X technology for barcoding and library preparation because human DNA apparently protects and enhances the recovery and integrity of bacterial DNA by unclear means [42].

**Figure 2:**
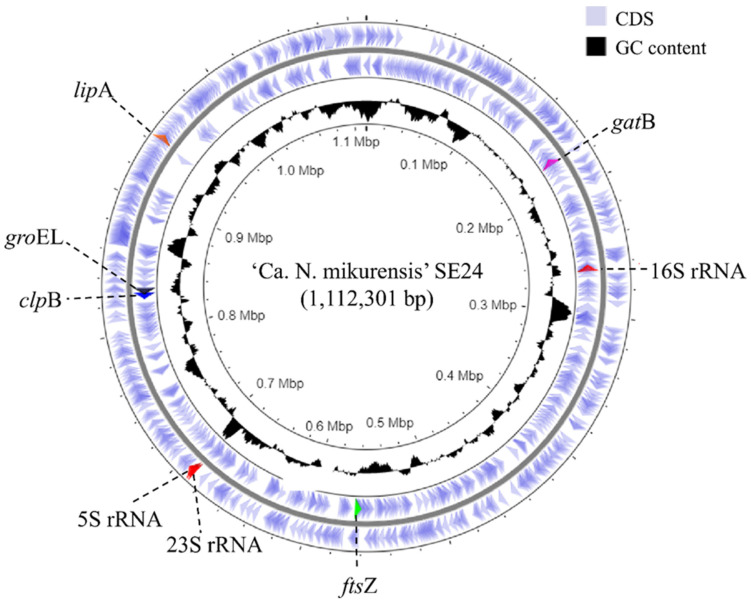
Circular representation of the chromosome of the reference genome of *Ca*. N. mikurensis strain SE24. From inside to outside, the first circle represents the genome size (Mbp), the second circle represents G + C contents (%), the third and fourth circles represent the coding sequences (CDS) on terminus and plus strands, respectively. The location of previously published MLSA genes and the complete ribosomal RNA genes (5S rRNA, 16S rRNA and 23S rRNA) within the genome are shown.

The SE24-1 plasma sample yielded the highest fraction of *Ca*. N. mikurensis DNA (5.08%), which probably reflects that this sample was extracted from fresh plasma, whereas the other plasma samples from the same patient (SE24-2 and SE24-3) were stored frozen at −120 °C before DNA extraction (Table 1). Moreover, this patient had a high bacterial burden as estimated by a recovery of 5.8 × 10^8^ *groEL* gene copies/mL blood, as determined by the diagnostic PCR (Table 1). This is equivalent to the number of bacteria/mL blood since the ‘*Ca*. N. mikurensis’ genome harbors only one copy of the *groEL* gene (Figure 2).

### 3.1. Genome Assembly and De Novo Annotation

The collected reads that did not map to the human whole-genome reference (HG19) were assembled using the 10× assembler Supernova and contigs were extracted at the megabubble level using limited linkage information whilst not fully collapsing the assembly into a pseudohaplotype. This resulted in 1008 contigs with 7 contigs >50 kb and a N50 (median contig size) 9801 of bp. The contigs were then analyzed by BLAST against the NCBI nucleotide BLAST-database and contigs with a hit against any *Ehrlichia* species were extracted. In total, 183 contigs were extracted and used as a basis for the draft assembly. Next, the contigs were aligned against themselves using the CLC Genome Finishing module and joined in multiple scaffolding rounds, reducing the total number of contigs to 85. The entire dataset was assembled again using Supernova, this time extracted at the Pseudohap level where the assembler creates a pseudohaplotype scaffold using linkage information and aligned against the clean contigs. This allowed us to join additional contigs and reduce the total number to 50, decreasing the assembly size to 1.14 Mbp. After genome finishing, the raw reads were mapped back to the assembly and the contig sequences were updated according to the mapping information. Initial annotation using Prokka revealed the complete sequences of five previously sequenced MLSA genes: 16S rRNA, *ftsZ*, *gatB*, *groEL* and *lipA*, as well as fragments of *clpB*; their spread-out positions within the genome indicated that the assembly was likely to contain the major part of the ‘*Ca*. N. mikurensis’ genome [17].

An additional sequencing run was performed with the goal of completing the genome of SE24. The new assembly was performed from the three sequenced SE24 samples using Supernova and again extracted at the megabubble level resulting in an assembly of 40,657 contigs. The dataset was reduced by eliminating contigs longer than 30 mbp and shorter than 4000 bp, obtaining 22,913 contigs that were analyzed by BLAST against the previous assembly and an existing reference genome of another *Ehrlichia* species, *Ehrlichia ruminantium* [43]. The BLAST results revealed a single contig of approximately 1 Mb containing the majority of the previous assembly sequence and another contig of 900 kb that contained the remaining parts. These two contigs were then joined using contig overlap. Further inspection revealed the new 2 Mb contig to contain two *Ca*. N. mikurensis genomes that could be split, producing a single contig of 1.11 Mb. The assembly indicated that the edges of the single contig contained a highly repetitive region, which prevented further assembly. Finally, the contig was corrected, first by using contigs from the original assembly, and subsequently by using mapping data and a majority vote where the nucleotide with the highest count at a given position was chosen.

The assembly annotation of the reference genome (SE24) yielded 900 genes in total, of which 860 represented coding sequences (CDS). Every tenth gene encoded a protein of unknown function (Table 2). The annotations contained all 6 MLSA-genes, 34 tRNA, as well as a complete ribosomal RNA operon (5S rRNA, 16S rRNA and 23S rRNA) (Table 2).

### 3.2. Intra-Species Genomic Comparisons

The SE20 and SE26 datasets were compared to the SE24 reference genome by mapping and variant calling analysis. SE26 differed by a mere 0.22‰ (245 single-nucleotide variants out of 1.1 million) and SE20 differed by 0.138‰ (153/1.1 million) with respect to SE24, respectively. The degree of genetic variation between the strains seemed to be in accordance with their mutual geographic distance, such that the variation was greatest between SE26 and SE24 and lower between SE20 and SE24 (Figure 1).

The proteins encoded by the three *Ca*. N. mikurensis strains (SE24, SE20 and SE26) were classified into functional Clusters of Orthologous Groups (COG) categories (Table 3). As expected, the majority of the genes’ encoded proteins were essential for bacterial survival, i.e., involved in bacterial biogenesis and replication; nutrient transport and metabolism. It is worth highlighting that close to every tenth protein of *Ca*. N. mikurensis was classified as “Function unknown” and 20% of the proteins did not assign to any COG category at all (Table 3).

**Figure 3:**
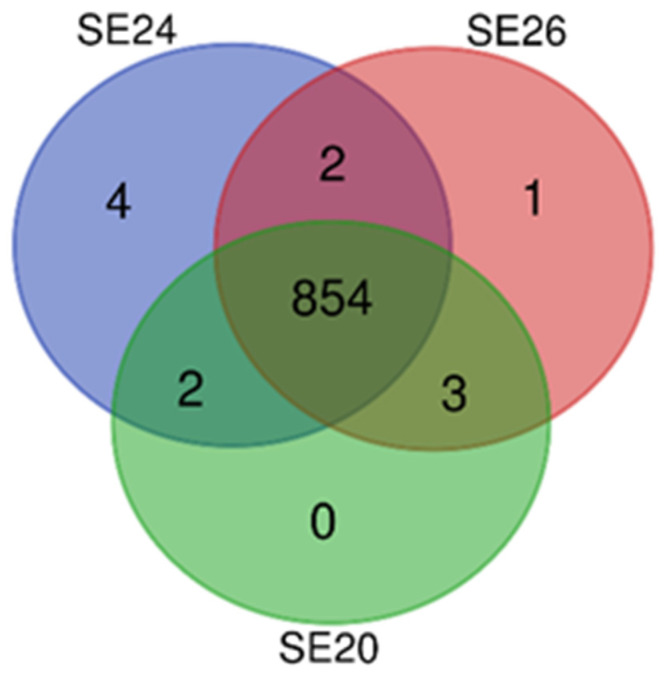
Venn diagram based on the consensus clusters of homologous proteins identified by the algorithms COGT and OrthoMCL. The predicted numbers of shared and unique protein-encoding genes derived from the three whole-genome-sequenced strains of *Ca*. N. mikurensis are shown.

Two of the strains were found to have exclusive proteins not shared by the other two strains: SE24 (4 exclusive proteins) and SE26 (1 exclusive protein) (Figure 3). One of the unique proteins of SE24 belonged to the P44/Msp-family (HL033_02985) and two were classified as hypothetical proteins with unknown function (HL033_02590, HL033_03370). However, the fourth hypothetical protein annotated by Prokka (between positions 53,155 and 63,249) was not annotated by PGAP. The hypothetical protein of strain SE24 that was encoded by HL033_02590 seemed to belong to the TrbC/VirB2 family according to BLASTP analysis. Studies has shown that VirB2 is a major pilus component of T4SS extracellular filaments and may play a critical role in the initial interaction with the host cell for members in the family *Anaplasmataceae* [45]. The unique protein of SE26 belonged to the thioredoxin-like family (IAH97_01635) and constitutes a small protein with antioxidant properties that is expressed in the periplasm or cytoplasm of certain Gram-negative bacterial species [46].

A search for prophages was inconclusive: Although a putative prophage of 13 kb was identified in all three strains, encompassing 6 genes in each case, the confidence score provided by Prophage Hunter software was low (0.56 to 0.63) rendering a verdict of “ambiguous”.

### 3.3. Comparison of the Ca. N. mikurensis Genome with Other Genomes within the Anaplasmataceae Family

We compared the reference genome of ‘*Ca*. N. mikurensis SE24′ with other whole-genome-sequenced members of the family *Anaplasmataceae* (Table 2). The genome of ‘*Ca*. N. mikurensis’ (1.11 Mb) was found to be the second smallest genome of the *Anaplasmataceae* family after *Neorickettsia sennetsu* (0.859 Mb) [26] with low GC contents typical of all members of the family except for *A. pagocytophilum* (41.6%). Low GC contents is a common trait of the genomes of intracellular bacteria [43]. ‘*Ca*. N. mikurensis’ and *E. ruminantium* contain almost the same number of pseudogenes (36 and 32, respectively), whereas *Anaplasma phagocytophilum* harbors four-fold more pseudogenes. Pseudogenes are genes that have become non-functional due to accumulation of mutations and are more frequent in intracellular bacteria where the loss of gene functions is compensated by bacterial parasitism on the host cell [44].

**Figure 4:**
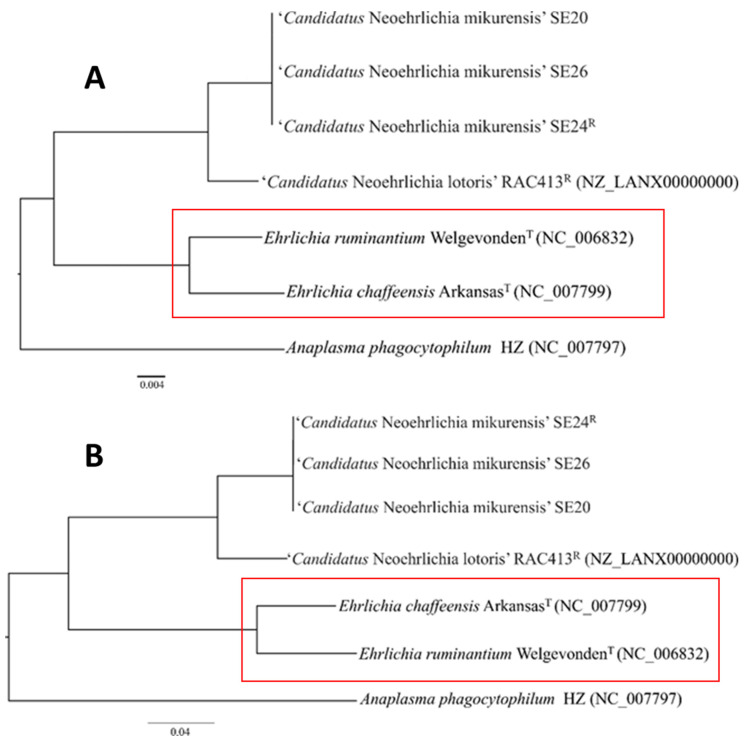
(**A**) Phylogenetic tree based on comparisons of the complete 16S rRNA gene sequences obtained from the genomes analyzed in this study. The scale bar indicates number of nucleotide subsitutions per site. (**B**) Core-genome dendrogram based on 93 shared core-proteins identified in this study. Marked in red is the switch in bacterial relatedness seen when a higher resolution phylogenetic analysis was used (**B)** vs. (**A**). The scale bar indicates aminoacid substitutions per site.

Phylogenetic analyses based on the complete 16S rRNA gene sequences determined ‘*Ca*. N. lotoris’ to be the most genetically related relative of ‘*Ca*. N. mikurensis’ and identified *E. ruminantium* as the most genetically related relative outside the genus of *Neoehrlichia* (Figure 4A) supporting earlier MLSA findings [17]. However, a higher-resolution phylogenetic analysis based on 93 sequenced core proteins showed that *Ca*. N. mikurensis is more closely related to the human pathogen *E. chaffeensis* than to *E. ruminantium* (Figure 4B). *E. chaffeensis* resembles *Ca*. N. mikurensis by being a human pathogen, in contrast to *E. ruminantium*, which is pathogenic for ruminants. In contrast, *Ca*. N. mikurensis and *E. ruminantium* share tropism for vascular endothelium, unlike *E. chaffeensis*, which preferentially infects monocytes [47].

All earlier sequenced members of the order *Rickettsiales* have a single rRNA operon in which the 16S rRNA gene is physically separated from the 23S-5S rRNA gene pair [43]. *Ca*. N. mikurensis also shares this feature, i.e., its 16S rRNA gene was found to be separated from the 23S-5S gene pair (Figure 2). Generally speaking, it is more common for bacteria to have multiple rRNA gene operons composed of genes located one after the other in the order of 16S-23S-5S [48,49]. The phenomenon of unlinked rRNA genes displayed by *Ca*. N. mikurensis seemed to be more frequent among slow-growing bacterial species and species that contained a single rRNA operon [50].

### 3.4. Protein Comparisons between Anaplasmataceae Species

**Figure 5:**
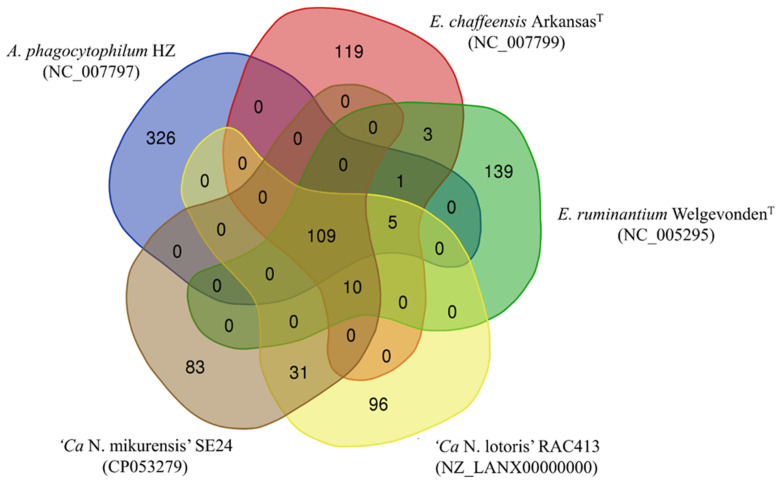
A consensus Venn diagram based on the clusters of homologous proteins detected by the algorithms COGT and OrthoMCL. The numbers of proteins predicted to be unique and shared, respectively, between *Ca*. N. mikurensis strain SE24 and four other bacterial species that are members of the family *Anaplasmataceae,* are shown.

Comparisons of the protein sets harbored by *Ca*. N. mikurensis with those of *A. phagocytophilum* HZ (GenBank accession no. CP000235), *E. chaffeensis* Arkansas^T^, *E. ruminatum* Welgenvonden^T^ and Ca. N. lotoris RAC-413 were done through a pan-genome approach. All species had 109 proteins in common, and ‘*Ca*. N. mikurensis’ harbored an additional 83 unique proteins not present in the other species. Further, 31 proteins were uniquely shared by ‘*Ca*. N. mikurensis’ and Ca. N. lotoris, and 10 proteins were shared by all species except for *A. phagocytophilum* (Figure 5). No proteins were solely shared by *Ca*. N. mikurensis and *A. phagocytophilum*, supporting their more distant relatedness compared with the other species.

A closer look at the ten proteins shared by ‘*Ca*. N. mikurensis’, Ca. N. lotoris, *E. ruminantium* and *E. chaffeensis,* but not by *A. phagocytophilum* (Table 4), showed that four are involved in translation and DNA repair, two in amino-acid biosynthesis, one in protein secretion, one in cellular detoxification, one in plasmid partitioning (although it is unclear if they possess plasmids) and one of unknown function. *Ehrlichia* species have a larger number of genes involved in amino acid biosynthesis compared with other members of the *Anaplasmataceae*-family, and it has been suggested that bacterial production of arginine may counter the host cell’s nitric oxide defense and allow the bacteria to weaken the host immune response [51].

All species share a gene *(prtD)* involved in secretion through the type I secretion system (T1SS), which enables many Gram-negative bacterial species to transport substrates from the bacterial cytosol to the extracellular space and also contributes to their virulence. *E.chaffeensis* secretes nucleomodulins able to reprogram host cell defense mechanisms and thereby facilitate bacterial invasion of host cells [52]. However, no shared genes were identified for ‘*Ca*. N. mikurensis’ and *E. ruminantium* that could explain their tropism for vascular endothelium.

To sum up, we have determined the complete genome sequence of ‘*Ca*. N. mikurensis’, which we hope will advance our understanding of the pathogenic mechanisms and immune evasion strategies employed by this emerging pathogen. Further, by combining proteomic analyses with the obtained genomic data, it may be possible in the near future to identify candidate outer-membrane proteins for the development of antibody assays to be used for diagnostics and seroepidemiologic studies, helping to determine the prevalence and incidence of this emerging infection in various populations and age groups.

## Figures and Tables

**Table 1:** Library statistics of *Ca*. N. mikurensis isolates sequenced from the blood plasma of three neoehrlichiosis patients (SE24, SE20, SE26).

Patient Sample	Number of Reads	Fraction of *Ca*. N. mikurensis DNA in Sequenced Plasma Sample (%)	Bacterial Load in Extracted Plasma (c/mL) ^a^
SE24-1	775,626,508	5.08	5.8 × 10^8^
SE24-2	729,557,012	0.10	5.8 × 10^8^
SE24-3	673,424,174	0.10	5.8 × 10^8^
SE20	748,875,060	1.25	1.2 × 10^8^
SE26	764,836,740	0.57	4.6 × 10^5^

^a^ Number of *groEL* gene copies/mL blood by diagnostic PCR. SE24-1, preparation from fresh blood sample. SE24-2 and SE24-3, frozen plasma preparations.

**Table 2:** Genome properties of *Ca*. N. mikurensis compared with that of other members of the family *Anaplasmataceae*.

Property	Organism				
	*Ca*. Neoehrlichia mikurensis SE24	*Ca*. Neoehrlichia lotoris	*Ehrlichia ruminantium*	*Ehrlichia chaffeensis*	*Anaplasma phagocytophilum*
Accession number	CP053279	NZ_LANX00000000	NC_005295	NC_007799	NC_007797
Size (bp)	1,112,301	1,268,660	1,516,355	1,176,248	1,471,282
GC content (%)	26.9	27.7	27.5	30.1	41.6
Genes, total (n)	900	953	987	965	1152
CDS, total (n)	860	912	944	922	1108
CDS with protein (n)	845	908	919	886	1105
Average CDS length (bp)	960	1016	1007	995	929
Assigned functions (n)	776	NR	758	604	747
Unknown functions (n)	90	NR	NR	85	77
Pseudogenes (n)	15	4	25	36	103
RNA genes (n)	40	41	43	43	44
rRNAs (n)	3	3	3	3	3
tRNAs (n)	34	35	36	37	37
ncRNAs (n)	3	3	4	3	4
Reference	This study	Daugherty, S.C et al. Direct submission	Collins et al. [44]	Dunning Hotopp et al. [26]	Dunning Hotopp et al. [26]

Bp, base pair; CDS, coding sequence; rRNA, ribosomal ribonucleic acid; tRNA, transfer ribonucleic acid; ncRNA, non-coding ribonucleic acid; NR, not recorded.

**Table 3:** Functional Clusters of Orthologous Groups of protein-coding genes from the three sequenced *Ca*. N. mikurensis strains.

Functional Category	*Ca*. N. mikurensis Strain SE24	*Ca*. N. mikurensis Strain SE20	*Ca*. N. mikurensis Strain SE26
	Number of Genes (%)		
Translation, ribosomal structure and biogenesis	112 (13)	115 (13)	115 (13)
Energy production and conversion	62 (7)	62 (7)	62 (7)
Posttranslational modification, protein turnover, chaperones	58 (7)	58 (7)	58 (7)
Coenzyme transport and metabolism	52 (6)	54 (6)	54 (6)
Replication, recombination and repair	47 (5)	47 (5)	47 (5)
Cell wall/membrane/envelope biogenesis	39 (5)	39 (5)	39 (5)
Nucleotide transport and metabolism	37 (4)	37 (4)	37 (4)
Inorganic ion transport and metabolism	34 (4)	34 (4)	34 (4)
Intracellular trafficking, secretion, and vesicular transport	32 (4)	32 (4)	32 (4)
Amino acid transport and metabolism	32 (4)	32 (4)	32 (4)
Lipid transport and metabolism	26 (3)	27 (3)	27 (3)
Transcription	23 (3)	23 (3)	23 (3)
Carbohydrate transport and metabolism	19 (2)	20 (2)	20 (2)
Cell cycle control, cell division, chromosome partitioning	11 (1)	11 (1)	11 (1)
Signal transduction mechanisms	10 (1)	10 (1)	10 (1)
Secondary metabolites biosynthesis, transport and catabolism	9 (1)	9 (1)	9 (1)
Defense mechanisms	3 (0.3)	3 (0.3)	3 (0.3)
General function prediction only	0	0	0
Mobilome: prophages, transposons	0	0	0
Cell motility	0	0	0
Cytoskeleton	0	0	0
Extracellular structures	0	0	0
RNA processing and modification	0	0	0
Chromatin structure and dynamics	0	0	0
Nuclear structure	0	0	0
Function unknown	90 (11)	90 (11)	90 (11)
No category assigned	170 (20)	166 (20)	167 (20)

Calculations done with eggNOG-mapper v2.

**Table 4:** Ten proteins predicted to be shared by Ca. Neoehrlichia mikurensis, Ca. Neoehrlichia lotoris, *Ehrlichia chaffeensis* and *Ehrlichia ruminantium*.

Protein	Function	Gene	Locus Tag
Argininosuccinate lyase	Amino-acid biosynthesis	*arg*H1	HL033_01080
Argininosuccinate synthase	Amino-acid biosynthesis	*arg*G	HL033_04485
ParA family protein	Partitioning of plasmids	*parA*	HL033_02250
Type I secretion system permease/ATPase	Protein secretion	*prtD*	HL033_03355
50S ribosomal protein L32	Translation	*rpm*F	HL033_03630
50S ribosomal protein L34	Translation	*rpm*H	HL033_03600
50S ribosomal protein L36	Translation	*rpm*J	HL033_04370
DNA repair protein RadA	DNA repair	*rad*A	HL033_02995
DUF2671 domain-containing protein	Protein with domain of unknown function	unknown	HL033_00465
Glutathione S-transferase family protein	Cellular detoxificaion	*gst*A	HL033_00805

## Data Availability

The sequencing data is available for public use at the sequence read archive of NCBI (http://www.ncbi.nlm.nih.gov/bioproject/PRJNA630882, accessed on 9 July 2021).
